# Feasibility evaluation of large language models in anesthesia-specific post-operative care instructions for total knee arthroplasty

**DOI:** 10.1016/j.pecinn.2025.100444

**Published:** 2025-11-22

**Authors:** Dhruv Nagesh, Donald P. Keating, Raghu V. Divakaruni, Bryan G. Beutel

**Affiliations:** Department of Primary Care, Kansas City University College of Medicine, Kansas City, MO, USA

**Keywords:** Large language model, Anesthesia, Post-operative, Instructions, Innovation, Arthroplasty, Patient education technology

## Abstract

**Objective:**

Large language models (LLMs) are increasingly applied in medicine, but their role in peri-operative education is underexplored. This pilot feasibility study compared four LLMs in producing post-operative care instructions for total knee arthroplasty (TKA).

**Methods:**

OpenAI GPT-4o, Claude 3.7 Sonnet, DeepSeek R1, and Gemini 2.0 Flash generated instructions from a standardized prompt. Outputs were scored (0 = does not meet, 1 = partially meets, 2 = fully meets) for accuracy, clarity, relevance, consistency, and readability. Accuracy was benchmarked against ERAS, ASA guidelines, and UpToDate. Readability was assessed using Flesch-Kincaid indices.

**Results:**

Within this limited sample, Claude, GPT-4o, and DeepSeek R1 demonstrated higher observed accuracy than Gemini, with Claude and GPT-4o showing full alignment with reference standards. Clarity scores were comparable across models. All achieved high relevance and internal consistency. Readability varied, with Gemini generating less readable text and GPT-4o and DeepSeek R1 producing more accessible content.

**Conclusion:**

LLMs can generate accurate, relevant, and consistent instructions, supporting their potential use in anesthesia education. Attention to readability and plain-language prompting may further enhance clinical utility.

**Innovation:**

This study provides one of the first anesthesia-specific evaluations of multiple LLMs, showing feasibility and opportunities for AI-driven patient communication.

## Highlights

• GPT-4o and Claude achieved perfect accuracy in TKA instructions.
• DeepSeek and GPT-4o produced the most readable outputs.
• Gemini scored lowest in both accuracy and readability.
• LLMs can support post-operative education in anesthesiology.
• Prompt design may further improve patient clarity and comprehension.

## 1 Introduction

Total knee arthroplasty (TKA) is among the most frequently performed orthopedic procedures, primarily indicated for end-stage osteoarthritis refractory to non-operative management. In the United States, approximately 790,000 TKAs are performed annually, with projections estimating 1.2 million procedures per year by 2030 [1,2]. While surgical advancements have improved peri-operative outcomes, the long-term success of TKAs also depends on effective post-operative care, including multimodal pain management, early mobilization, and prevention of complications such as venous thromboembolism and joint stiffness [[3], [4], [5]]. A crucial component of post-operative recovery is patient education, which ensures adherence to activity restrictions, pain control strategies, and physical therapy protocols. However, several barriers can impair the delivery and retention of post-operative instructions. Post-anesthesia cognitive dysfunction is common and time constraints during discharge planning often limit the quantity and quality of education provided [6,7]. Furthermore, patients exhibit variability in health literacy, and instructions are frequently written above recommended readability levels, hindering comprehension and adherence [8,9]. These challenges highlight the need for innovative approaches to improve post-operative education delivery.

Large language models (LLMs) offer a promising solution. LLMs use generative artificial intelligence (AI) to produce coherent, contextually relevant human-like responses in a process called natural language processing (NLP). Popular models, such as OpenAI's ChatGPT-4o or Google's Gemini, are being explored for medical applications, including summarizing clinical content, explaining treatment options, and answering patient questions [10,11]. Their ability to simplify medical information and tailor responses to a user's comprehension level presents a unique opportunity to improve patient understanding, particularly in post-operative settings where education is critical to recovery. Unlike static written materials, LLMs can deliver interactive, personalized explanations that may enhance retention and engagement [12,13].

For TKA patients, this adaptability could address varying needs across different recovery phases, activity levels, and pain experiences. Prior studies have investigated generative post-operative NLP output, but none have directly assessed TKA. For instance, ChatGPT-4 has been evaluated for producing post-operative care instructions following tonsillectomy, demonstrating its potential in otolaryngology settings [14]. Similarly, LLM-generated discharge summaries have been evaluated in emergency settings, which emphasized improvements in readability and patient comprehension [15]. Additionally, research has explored the application of ChatGPT in enhancing post-operative guidance for tympanoplasty patients, highlighting its role in improving patient education and adherence [16]. The present study aimed to evaluate and compare the performance of four LLMs (GPT-4o, Claude, Gemini, and DeepSeek R1) in generating post-operative care instructions for patients undergoing TKA under general anesthesia. The measurable outcomes of this investigation included medical accuracy, clarity, relevance, consistency, and readability to determine whether LLM-generated content meets both clinical standards and patient communication needs.

## 2 Methods

### 2.1 Model selection and prompting

Four widely-available LLMs were evaluated for their ability to generate post-operative care instructions for TKA: OpenAI GPT-4o (model identifier: gpt-4o-mini-2024-10-01, accessed October 12, 2024), Anthropic Claude 3.7 Sonnet (accessed October 13, 2024), Google Gemini 2.0 Flash (accessed October 15, 2024), and DeepSeek R1 (DeepSeek AI, accessed February 2, 2025). These LLMs were specifically chosen due to their popularity within the United States, with approximately 52 % of U.S. adults having used them [17]. DeepSeek R1, the newest product on the market, offers similar functionality, providing users with interactive, conversational exchanges across various topics. Claude, Gemini, and GPT-4o were evaluated in October 2024, while DeepSeek R1 was evaluated in February 2025. Because model releases occurred at different times, outputs were collected using the most current publicly available versions at each date. Each model was given a standardized prompt requesting detailed instructions for a patient recovering from TKA under general anesthesia, with emphasis on pain management, common side effects, activity restrictions, and early mobilization. The prompt was as follows:

*“Generate detailed post-operative care instructions for a patient who has undergone general anesthesia for total knee arthroplasty, focusing on pain management, common side effects, activity restrictions, and mobilization.”*

Each model was prompted three consecutive times using identical wording to generate three independent outputs. No outputs were manually regenerated or excluded to avoid selection bias. All models were accessed through their publicly available interfaces using default settings to ensure comparability and reproducibility.

### 2.2 Evaluation domains

Three responses were collected from each model (for a total of 12 responses; see Supplementary 1) and evaluated across five distinct domains: medical accuracy, clarity, relevance to TKA-specific recovery, consistency, and readability (Table 1). Scoring was conducted using a three-point scale, where 0 indicated non-compliance with recommendations, 1 indicated partial adherence, and 2 indicated full alignment with established guidelines. Scoring was performed independently by two separate investigators. Two investigators independently scored each model output across all domains and subsequently met to review any discrepancies. Final scores were determined by consensus to ensure uniform application of evaluation criteria. Because ratings were reconciled by consensus, statistical inter-rater reliability metrics (e.g., ICC or κ) were not calculated. Considering this pilot investigation aimed to evaluate reproducibility and reliability of LLM outputs in a controlled, single-prompt context, each model was prompted three times using an identical prompt to assess internal consistency rather than inter-prompt variability.

With respect to each domain, medical accuracy was assessed based upon adherence to Enhanced Recovery After Surgery (ERAS) and American Society of Anesthesiologists (ASA) guidelines, as well as UpToDate recommendations, with particular attention to evidence-based pain management strategies, appropriate side effect management (e.g., nausea, constipation), and promotion of early mobilization [[18], [19], [20], [21]]. Clarity was evaluated by the use of simple, jargon-free language, clear explanations of medical terms, logically structured action steps, and a supportive, instructive tone. Relevance was determined by the extent to which responses addressed TKA-specific post-operative needs, such as joint mobility, weight-bearing status, management of pain and swelling, and the exclusion of unrelated or general surgical details. Consistency was defined as the uniformity of recommendations across each model's three outputs, particularly in core areas such as pain management, side effect mitigation, and mobilization instructions. Readability was assessed using the Flesch Reading Ease Score and Flesch–Kincaid Grade Level, calculated directly from each model's complete generated output (three per model). Each index was derived using the standard Flesch–Kincaid formulas based on average sentence length and syllables per word, with an emphasis on achieving a sixth-to-eighth-grade reading level while maintaining sentence clarity and accessibility for general patient populations [22]. A Flesch Reading Ease Score between 80 and 90 aligns with a sixth-to-eighth grade reading level, making it suitable for general audiences. Higher scores indicate greater readability while lower scores suggest more challenging text.

**Table 1:** Domains, scoring scale, and operational criteria used to evaluate large language model post-operative care instructions for total knee arthroplasty. Abbreviations: ASA, American Society of Anesthesiologists; ERAS, Enhanced Recovery After Surgery; F-K, Flesch–Kincaid; TKA, total knee arthroplasty.

Domain	Scale	Criteria
Medical Accuracy	0–2	Pain management aligns with ERAS and ASA guidelines Side effects (nausea, dizziness) align with ASA guidelines Includes early mobilization recommendations as per ERAS protocols Overall aligns well with established TKA post-op guidelines
Clarity / Patient-Friendliness	0–2	Uses simple language without excessive medical jargon Terms explained in patient-friendly manner Instructions broken into actionable steps Overall tone is supportive and easy to follow
Relevance to TKA Post-Op Care	0–2	Focus on TKA-specific recovery needs (joint mobility, weight-bearing restrictions) Addresses swelling, pain, and TKA-relevant side effects Avoids unrelated surgical care details
Consistency Across Outputs	0–2	Provides similar recommendations across all three outputs No significant contradictions or major variations Key areas (pain, side effects, mobilization) remain consistent
Readability	Continuous	F-K Grade Level (target 6–8) Flesch Reading Ease (higher = easier)

Institutional review board approval was not required for this study as it involved publicly-available LLMs without any human or animal subjects or their data.

### 2.3 Statistical analysis

Descriptive statistics (mean ± standard deviation) were used to summarize domain scores across models. Because the scoring scale for accuracy, clarity, and relevance was ordinal (0–2), these data were analyzed descriptively rather than as continuous variables. Non-parametric Kruskal–Wallis tests with Dunn's post-hoc comparisons were conducted only as exploratory checks to visualize potential trends, recognizing that the small sample size (three outputs per model) precludes inferential conclusions.

Given the pilot and feasibility nature of this study, an a priori sample-size calculation was not performed, and statistical testing was used descriptively to illustrate observed trends rather than to draw definitive comparative conclusions.

## 3 Results

### 3.1 Accuracy

**Fig. 1:**
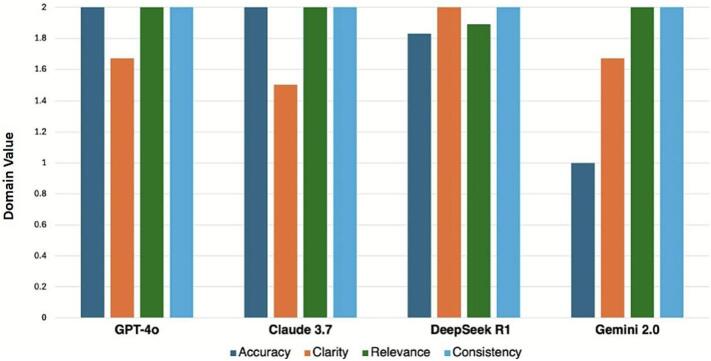
LLM performance across qualitative domains. Bar graph comparing performance scores across four large language models (GPT-4o, Claude 3.7 Sonnet, DeepSeek R1, and Gemini 2.0 Flash) in four qualitative domains: medical accuracy, clarity, relevance, and consistency. Abbreviations: LLM = large language model; GPT = Generative Pre-trained Transformer.

As noted in Table 2, Claude and GPT-4o demonstrated higher observed accuracy scores than Gemini within this small sample, each achieving full alignment (2.00 ± 0.00) (Fig. 1). DeepSeek R1 (1.83 ± 0.14) also showed higher accuracy than Gemini (1.00 ± 0.00), while scores among DeepSeek R1, Claude, and GPT-4o were similar.

**Table 2:** Comparative performance of four large language models across qualitative and readability domains.

LLM	Accuracy	Clarity	Relevance	Consistency	Grade Level	Reading Ease
*GPT-4o*	2.00 ± 0.00	1.67 ± 0.29	2.00 ± 0.00	2.00 ± 0.00	9.40 ± 0.20	44.80 ± 0.46
*Claude 3.7*	2.00 ± 0.00	1.50 ± 0.00	2.00 ± 0.00	2.00 ± 0.00	9.20 ± 0.69	38.10 ± 4.44
*DeepSeek R1*	1.83 ± 0.14	2.00 ± 0.00	1.89 ± 0.19	2.00 ± 0.00	9.00 ± 0.55	43.93 ± 4.12
*Gemini 2.0*	1.00 ± 0.00	1.67 ± 0.29	2.00 ± 0.00	2.00 ± 0.00	10.67 ± 0.40	37.80 ± 4.05

Data are represented as mean ± standard deviation, with three prompts per model. Abbreviations: LLM, large language model.

### 3.2 Clarity

No meaningful differences in clarity scores were observed among the models. Gemini (1.67 ± 0.29), GPT-4o (1.67 ± 0.29), Claude (1.50 ± 0.00), and DeepSeek R1 (2.00 ± 0.00) produced similarly clear and well-structured responses.

### 3.3 Relevance

Gemini, Claude, and GPT-4o each achieved top relevance scores (2.00 ± 0.00). DeepSeek R1 scored slightly lower (1.89 ± 0.19), but this difference was minor within the limited sample.

### 3.4 Consistency

All four models achieved ideal consistency (2.00 ± 0.00), showing full agreement across their three outputs in key areas such as pain management, mobilization, and side-effect recommendations.

### 3.5 Readability

As reflected in Table 3, exploratory analyses suggested variability in readability across models. Gemini (10.67 ± 0.40) generated text at a higher grade level (less readable) than Claude (9.20 ± 0.69), GPT-4o (9.40 ± 0.20), and DeepSeek R1 (9.00 ± 0.55). Differences among Claude, GPT-4o, and DeepSeek R1 were small. Flesch Reading Ease scores followed a similar pattern, with GPT-4o (44.80 ± 0.46) and DeepSeek R1 (43.93 ± 4.12) showing greater readability than Gemini (37.80 ± 4.05) and Claude (38.10 ± 4.44).

**Table 3:** Scoring of readability grade level and ease score across generated large language model post-operative care instructions.

LLM	Readability	Response 1	Response 2	Response 3	Mean ± Standard Deviation
Gemini	Grade Level	11.0	10.2	10.8	10.67 ± 0.40
Claude		8.8	10.0	8.8	9.20 ± 0.69
GPT-4o		9.6	9.4	9.2	9.40 ± 0.20
DeepSeek R1		8.9	9.6	8.5	9.00 ± 0.55
Gemini	Ease Score	35.1	42.7	35.6	37.80 ± 4.05
Claude		40.9	32.4	41.0	38.10 ± 4.44
GPT-4o		44.4	44.7	45.3	44.80 ± 0.46
DeepSeek R1		46.0	38.8	47.0	43.93 ± 4.12

Abbreviations: LLM, large language model.

## 4 Discussion and conclusion

### 4.1 Discussion

The long-term success of total knee arthroplasty (TKA) depends on overcoming barriers such as post-anesthesia cognitive impairments, which necessitate innovative and personalized approaches to patient instruction. Large language models (LLMs) represent a promising innovation given their ability to generate accurate educational content and answer foundational medical questions with high precision [23]. However, their reliability declines sharply when prompted to apply their knowledge in clinically complex or highly specialized contexts [24]. Moreover, considerable variability in performance exists across different LLMs, even within the same field, raising potential safety concerns [25]. This study addressed this gap by evaluating the capabilities of four leading LLMs (GPT-4o, Claude, DeepSeek R1, and Gemini) in generating post-operative care instructions tailored to patients undergoing TKA under general anesthesia. The findings highlight both the potential and current limitations of LLMs in supporting patient education during recovery. Because the scoring data were ordinal and the number of model outputs was limited, all statistical analyses were interpreted descriptively, and findings should be viewed as exploratory trends rather than inferential results.

#### 4.1.1 Model performance and interpretation

Claude and GPT-4o were notable for their medical accuracy, closely mirroring key elements from established peri-operative guidelines, including multimodal pain strategies, mobilization timing, and side effect mitigation. Their alignment with standards such as those from the ASA, UpToDate, and ERAS protocols underscores their potential reliability as educational tools. Gemini's outputs demonstrated lower alignment with clinical reference standards compared with the other models. The reasons for this difference are uncertain and may relate to variations in model design, dataset composition, or optimization, although these factors were not directly examined in this study. The quality and scope of training data are known to influence LLM performance in general, but specific causes for the observed variation cannot be determined here. Google has announced the Med-Gemini model, which incorporates domain-specific biomedical data; however, its performance was not evaluated in this feasibility study and remains an area for future investigation [26]. Furthermore, while clarity and relevance were consistent across models, DeepSeek R1 showed minor gaps in procedure-specific content. This suggests that even well-performing models may benefit from fine-tuning to consistently capture the nuances of individual surgical pathways. The most pronounced divergence occurred in readability: Gemini's outputs were notably more complex, potentially limiting its accessibility to patients with average or low health literacy [27]. In contrast, GPT-4o and DeepSeek R1 consistently generated simpler, more digestible language which aligns better with patient education standards. While several models demonstrated excellent accuracy and relevance, their readability scores exceeded the sixth-to-eighth-grade level typically recommended for patient-facing materials. This finding underscores that accuracy alone does not guarantee effective communication. Excessively complex language can hinder comprehension and adherence, particularly among patients with limited health literacy. Improving readability through optimized prompting or fine-tuning for plain-language outputs may enhance the practical utility of LLM-generated educational materials without sacrificing medical accuracy.

Our findings broadly align with and expand upon prior studies assessing the performance of LLMs in medical communication. Swisher et al. evaluated ChatGPT's ability to revise educational materials from the American Rhinologic Society and the American Academy of Facial Plastic and Reconstructive Surgery [12]. They found ChatGPT-4 significantly improved readability without compromising quality, consistent with our observation that GPT-4o and DeepSeek R1 produced outputs within recommended patient reading levels, outperforming Gemini in this domain. Similarly, Vallurupalli et al. assessed ChatGPT 3.5 in the context of craniofacial education materials [13]. Their study reported improved readability and preservation of medical accuracy, reflecting our own findings for GPT-4o and Claude, which scored highly in both clarity and medical accuracy. These parallels suggest that more advanced LLMs may be capable of producing content that is both understandable and clinically relevant. Ratnagandhi et al. directly compared GPT-4, Claude, and Gemini in anesthetic patient education [28]. The study showed that Gemini ranked highest in readability, GPT-4 was most accurate, and Claude delivered the most comprehensive content. This contrasts with our findings, where Gemini produced the least readable outputs and underperformed in medical accuracy. The discrepancy may be due to differences in model versions (e.g. Gemini Flash 2.0 versus earlier), prompt specificity, or evaluation criteria that emphasize the variability in LLM performance across contexts and versions.

#### 4.1.2 Clinical implications

The integration of LLMs into post-operative education workflows offers a new paradigm for patient communication [28]. Unlike conventional handouts or scripted instructions, LLMs are capable of generating responsive and context-dependent guidance. This adaptability could prove useful during discharge, when time is constrained and patients may be cognitively impaired or overwhelmed. Considering this, one potential promising application is embedding LLMs within electronic health record systems to generate tailored post-operative summaries or educational pamphlets. This integration could streamline provider workflow and ensure that instructions are personalized and current. However, variability among models raises questions about standardization and oversight. Without appropriate quality control or model selection, there is a risk of delivering inconsistent or complex instructions with the potential to negatively affect adherence. These tools must be treated not as standalone replacements, but as augmentative supports that can be optimized through prompt engineering and clinical validation. Additionally, future implementation must consider practical barriers such as cost, technical integration with hospital systems, and regulatory oversight. Addressing these challenges will be key to enabling scalable, safe, and equitable use of LLMs in practice.

#### 4.1.3 Limitations

This study has several important limitations:

1. Sample size and design: With three generations per model for a single standardized prompt, the study is best interpreted as a reliability assessment of intra-model consistency rather than a full variability analysis. The small sample size provides adequate power to detect large effects, but more modest differences may have gone undetected.
2. Temporal non-concurrency: GPT-4o, Claude, and Gemini were evaluated in October 2024, whereas DeepSeek R1 was tested in February 2025. Because LLMs are frequently updated, model-drift bias or retraining during this interval could have affected comparative performance. Future work should freeze model identifiers and test contemporaneously.
3. Model selection: Only general-purpose, publicly available models were examined. Clinically-tuned or health-specific versions were not accessible at the time of testing. Including such models in future research could clarify the benefits of medical fine-tuning.
4. Patient-level validation: The study did not include direct patient testing or outcomes data, so real-world comprehension and adherence effects remain unknown [29].
5. Evaluation process: Outputs were scored by two reviewers who reached consensus on final ratings. Because consensus scores were used, formal inter-rater reliability statistics were not calculated. Future studies should retain independent ratings to quantify agreement.
6. Training-data transparency: The evaluated models rely on proprietary, non-open-source data, introducing potential bias and limiting reproducibility.
7. Scope of models: While several leading systems were analyzed, the selection was not exhaustive, and other models trained on domain-specific datasets may yield different results.

Despite these limitations, this pilot work provides an early framework for evaluating LLMs in anesthesia-specific patient education and highlights key considerations for future, higher-powered studies.

### 4.2 Innovation

This study provides one of the first structured evaluations of LLMs in anesthesia-specific post-operative education. By benchmarking outputs from multiple leading LLMs against perioperative guidelines for TKAs, we introduce a reproducible framework for assessing accuracy, clarity, and readability in a procedure-specific context. The work demonstrates the feasibility of integrating LLMs as supplementary educational tools in anesthesiology and highlights their potential to standardize discharge communication and improve patient comprehension. These findings serve as a proof-of-concept for responsible AI integration into perioperative care and future studies on optimizing readability and clinical validation.

### 4.3 Conclusion

LLMs such as GPT-4o and Claude demonstrate high potential for enhancing post-operative education following TKA, particularly in delivering accurate and accessible instructions. DeepSeek R1 also performed well in readability and general clarity, whereas Gemini lagged in both accuracy and readability. While the findings support the utility of LLMs as supplementary educational tools, variability in output highlights the need for further refinement before widespread clinical adoption. As LLMs evolve, their thoughtful integration into discharge processes could help address gaps in health literacy, support adherence, and ultimately improve surgical recovery outcomes.

The following is the supplementary data related to this article.

Supplementary 1 Generated outputs from each of the four LLMs. Supplementary 1

## Consent to participate / consent for publication

Not applicable.

## CRediT authorship contribution statement

**Dhruv Nagesh:** Writing – review & editing, Writing – original draft, Resources, Project administration, Methodology, Investigation, Formal analysis, Data curation, Conceptualization. **Donald P. Keating:** Writing – review & editing, Writing – original draft, Methodology, Investigation, Formal analysis, Data curation, Conceptualization. **Raghu V. Divakaruni:** Writing – review & editing, Writing – original draft, Methodology, Investigation, Formal analysis, Data curation. **Bryan G. Beutel:** Writing – review & editing, Writing – original draft, Supervision.

## Ethical approval

This study did not involve human participants or animals and was therefore exempt from institutional review board approval.

## Funding

This research did not receive any specific grant from funding agencies in the public, commercial, or not-for-profit sectors.

## Declaration of competing interest

The authors declare that they have no known competing financial interests or personal relationships that could have appeared to influence the work reported in this paper.

## Glossary

LLM (Large Language Model): Artificial intelligence system trained on vast datasets to generate human-like text.
NLP (Natural Language Processing): A field of Artificial Intelligence focused on enabling computers to understand and produce human language.
TKA (Total Knee Arthroplasty): A surgical procedure, also known as knee replacement, performed to relieve pain and restore function in patients with advanced knee disease.
ERAS (Enhanced Recovery After Surgery): Evidence-based peri-operative protocols designed to improve patient outcomes and speed recovery.
ASA (American Society of Anesthesiologists): Professional organization that develops guidelines and standards for anesthesia practice.
Flesch Reading Ease Score: A readability metric; higher scores indicate text is easier to read.
Flesch-Kincaid Grade Level: A readability formula estimating the U.S. school grade required to understand a text.
