# Supplementary material for: Feasibility evaluation of large language models in anesthesia-specific post-operative care instructions for total knee arthroplasty
Source: PEC Innov. 2025 Nov 22;8:100444. doi: 10.1016/j.pecinn.2025.100444 (PMC12704057; doi:10.1016/j.pecinn.2025.100444)
Supplement: Supplementary 1 — Generated outputs from each of the four LLMs. [file mmc1.docx]

## **Gemini 2.0 Flash Output #1**

## **Post-Operative Care Instructions for Total Knee Arthroplasty**

Congratulations on completing your total knee arthroplasty (knee replacement)! This surgery is a significant step towards improving your quality of life. To ensure a successful recovery, please follow these post-operative care instructions:

### **Pain Management**

- Medication: As prescribed by your doctor, take pain medication as directed. This may include prescription pain relievers, over-the-counter medications, or a combination of both.
- Ice: Apply ice packs to your knee for 20-minute intervals every 2-3 hours. This can help reduce swelling and pain.
- Elevation: Keep your leg elevated on pillows when resting to help with swelling.

### **Potential Side Effects**

- Swelling: It is normal for your knee to be swollen after surgery. This should gradually improve over time.
- Pain: You may experience some pain, even with medication. This is also normal.
- Blood Clots: To reduce the risk of blood clots, follow your doctor's instructions regarding blood thinners and leg exercises.
- Infection: Keep your incision clean and dry. Report any signs of infection, such as redness, swelling, warmth, or drainage, to your doctor immediately.

### **Activity Restrictions**

- Weight-bearing: Your doctor will provide specific instructions on when you can begin putting weight on your knee. Initially, you may be instructed to use crutches or a walker.
- Range of Motion: Follow your physical therapist's guidance for gentle range of motion exercises to prevent stiffness and improve flexibility.
- Avoid Activities: Avoid activities that put excessive stress on your knee, such as running, jumping, or squatting.

### **Importance of Early Mobilization**

- Prevent Complications: Early mobilization helps prevent blood clots, stiffness, and muscle atrophy.
- Improve Recovery: By following your physical therapist's guidance and participating in rehabilitation exercises, you can speed up your recovery process.

Remember: Your recovery is a journey, and progress may vary. If you have any concerns or questions, please do not hesitate to contact your healthcare provider.

##

## **Gemini 2.0 Flash Output #2**

## **Post-Operative Care Instructions for Total Knee Arthroplasty**

Congratulations on completing your total knee arthroplasty (knee replacement)! This surgery is a significant step towards improving your quality of life. To ensure a successful recovery, please follow these post-operative care instructions:

### **Pain Management**

- Medication: As prescribed by your doctor, take your pain medication as directed. This may include prescription pain relievers, over-the-counter medications, or a combination of both.
- Ice: Apply ice to your knee for 20-minute intervals every 2-3 hours to reduce swelling and pain. Use a thin cloth between the ice and your skin to protect your skin.

### **Potential Side Effects**

- Swelling: It's normal for your knee to be swollen after surgery. This swelling should gradually decrease over time.
- Pain: You may experience some pain, even with medication. This is normal, but if the pain becomes severe or unbearable, contact your doctor immediately.
- Bleeding: Some minor bleeding or oozing from the incision is expected. However, if you notice excessive bleeding or soaking through multiple dressings, contact your doctor.
- Blood clots: Deep vein thrombosis (DVT) is a serious condition that can occur after surgery. To reduce your risk, follow your doctor's instructions regarding blood thinners and leg exercises.

### **Activity Restrictions**

- Weight-bearing: Your doctor will provide specific instructions on when you can begin putting weight on your knee. Initially, you may be instructed to use crutches or a walker.
- Range of motion: Gentle range-of-motion exercises will be prescribed to help restore movement in your knee. Follow your physical therapist's guidance.
- Avoid strenuous activity: Avoid activities that put excessive stress on your knee, such as running, jumping, or high-impact sports.

### **Importance of Early Mobilization**

- Prevent complications: Early mobilization helps prevent complications such as blood clots and stiffness.
- Improve healing: Movement promotes blood flow to the area, which can help your knee heal more quickly.
- Restore function: Regular exercise will help you regain strength and flexibility in your knee.

Remember, it's essential to follow your doctor's specific instructions and attend all scheduled physical therapy appointments. By doing so, you can optimize your recovery and enjoy the benefits of your new knee joint. If you have any questions or concerns, please don't hesitate to contact your doctor or healthcare provider.

## **Gemini 2.0 Flash Output #3**

## **Post-Operative Care Instructions for Total Knee Arthroplasty**

**Pain Management:**

- Medication: Follow your doctor's instructions for taking prescribed pain medication. Do not stop taking medication without consulting your doctor, even if you feel better.
- Ice and Heat: Apply ice to your knee for 20 minutes every hour for the first few days. After the initial swelling subsides, you can alternate between ice and heat.
- Elevation: Keep your leg elevated on pillows when resting to reduce swelling.

**Potential Side Effects:**

- Swelling: This is normal after surgery. It should gradually improve over time.
- Pain: While pain medication will help, some discomfort is expected. Notify your doctor if the pain becomes severe or unmanageable.
- Blood Clots: To reduce the risk of blood clots, follow your doctor's instructions regarding blood thinners and leg exercises.
- Infection: Keep your incision clean and dry. Notify your doctor immediately if you notice any signs of infection, such as redness, swelling, warmth, or drainage.

**Activity Restrictions:**

- Weight-Bearing: Your doctor will provide specific instructions on when and how much weight you can put on your knee. Initially, you may be instructed to use crutches or a walker.
- Range of Motion: Follow your physical therapist's guidance for gentle range of motion exercises to improve flexibility.
- Avoid Activities: Avoid activities that put excessive stress on your knee, such as running, jumping, or squatting.

**Importance of Early Mobilization:**

- Prevent Stiffness: Early movement helps prevent stiffness and promotes healing.
- Strengthen Muscles: Gentle exercises help strengthen the muscles surrounding your knee.
- Improve Function: Early mobilization is essential for restoring normal knee function.

**Follow-Up Care:**

- Regular Check-ups: Schedule follow-up appointments with your doctor as instructed.
- Physical Therapy: Attend all physical therapy sessions to learn proper exercises and techniques.
- Report Changes: If you experience any new or worsening symptoms, contact your doctor immediately.

Remember, these instructions are general. Your specific care plan may vary based on your individual situation. Always follow your doctor's advice and recommendations.

#

# **Claude 3.7 Sonnet Output #1**

# **Post-Operative Care Instructions: Total Knee Arthroplasty**

## **Medication Pain Management**

- Take prescribed pain medications as directed by your surgeon
- Typical medications include:
  - Prescription pain medication (opioids) for severe pain
  - NSAIDs (if approved by your surgeon) for inflammation
  - Acetaminophen for mild to moderate pain
- Keep track of all medications taken
- Do not exceed recommended dosages
- Take medications before pain becomes severe

### **Non-Medication Pain Management**

- Ice therapy: 20 minutes on, 20 minutes off
- Elevation: Keep knee above heart level when resting
- Compression: Use prescribed compression stockings
- Rest: Get adequate sleep and take breaks between exercises

## **Activity Guidelines**

### First 24 Hours

- Rest frequently but do not stay completely immobile
- Begin ankle pumps and gentle knee flexion exercises as directed
- Use walker or crutches for all movement
- Do not shower (keep incision dry)

### Days 2-14

- Follow physical therapy protocol exactly
- Gradually increase walking distance with proper assistive device
- Continue prescribed exercises 2-3 times daily
- Maintain proper wound care and dressing changes
- Begin showering once approved by surgeon (typically 5-7 days)

### Weeks 2-6

- Progress to cane or single crutch when directed by physical therapist
- Increase exercise intensity as tolerated
- Resume light household activities
- No driving until cleared by surgeon
- Continue wound care until fully healed

## **Movement Restrictions**

### DO NOT:

- Pivot or twist on operated leg
- Kneel on operated knee
- Cross legs while sitting or lying
- Sit in low chairs or sofas
- Carry heavy objects
- Push through sharp or severe pain

### DO:

- Use assistive devices as prescribed
- Keep leg in line with body during movement
- Use proper body mechanics for sitting/standing
- Follow weight-bearing restrictions as specified

## **Warning Signs**

Seek immediate medical attention if you experience:

- Fever over 101.5°F (38.6°C)
- Severe, uncontrolled pain
- Excessive redness, warmth, or drainage from incision
- Calf pain or swelling
- Chest pain or shortness of breath
- Opening of surgical wound

## **Physical Therapy Schedule**

- Begin in-hospital PT day of or day after surgery
- Continue PT exercises at home as prescribed
- Attend outpatient PT sessions as scheduled
- Typical schedule: 2-3 times per week for 6-8 weeks

## **Daily Living Modifications**

### Bathroom Safety

- Use elevated toilet seat
- Install grab bars if needed
- Use long-handled sponge for bathing
- Shower chair when approved to shower

### Home Setup

- Remove throw rugs and potential tripping hazards
- Arrange furniture for clear walkways
- Keep frequently used items at waist level
- Prepare meals ahead or arrange for assistance

## **Recovery Milestones**

### Week 1-2

- Walk with walker/crutches
- Achieve 0-90 degrees knee flexion
- Independent with basic self-care

### Week 3-6

- Progress to cane/single crutch
- Increase walking distance
- Improve range of motion
- Resume light household activities

### Week 6-12

- Walk without assistive device
- Return to light work duties
- Drive (if surgery was on right knee)
- Continue strengthening exercises

## **Follow-up Care**

- First post-op visit: 10-14 days
- Second visit: 4-6 weeks
- Third visit: 3 months
- Annual check-ups thereafter

## **Contact Information**

Keep these numbers readily available:

- Surgeon's office
- Physical therapy clinic
- Emergency contact
- Local emergency room

Remember: Early mobilization and proper adherence to these instructions are crucial for optimal recovery. Each patient's recovery may vary; always follow your surgeon's specific instructions if they differ from these general guidelines.

#

# **Claude 3.7 Sonnet Output #2**

# **Post-Operative Care Instructions: Total Knee Arthroplasty**

### **Medication Schedule Pain Management**

- Take prescribed pain medications as directed by your surgeon
- Typical medications include:
  - Prescription pain medication (opioids) for severe pain
  - NSAIDs (if approved by your surgeon) for inflammation
  - Acetaminophen for mild pain
- Take medications with food to prevent stomach upset
- Do not exceed recommended dosages

### **Non-Medication Pain Management**

- Ice therapy: 20 minutes on, 20 minutes off
- Elevation: Keep knee above heart level when resting
- Compression: Use prescribed compression stockings as directed
- Rest: Get adequate sleep and take breaks between exercises

## **Activity Guidelines**

### First 24-48 Hours

- Start ankle pumps immediately to promote circulation
- Begin gentle knee flexion and extension exercises as directed
- Use walker or crutches for all movement
- Do not attempt to walk without assistance

### First Two Weeks

- Progress to weight-bearing as tolerated unless otherwise directed
- Perform prescribed exercises 3-4 times daily
- Gradually increase walking distance
- Continue using walking aids until cleared by physical therapist
- No driving while taking narcotic pain medication

### Weeks 2-6

- Continue physical therapy exercises
- Progress to cane or single crutch when advised
- May resume light household activities
- No kneeling, squatting, or high-impact activities

## **Early Mobilization**

### Exercise Program

1. Ankle pumps: 10 repetitions every hour while awake
2. Knee straightening exercises
   - Quad sets: Hold 5-10 seconds, 10 repetitions
   - Straight leg raises: 10 repetitions, 3 times daily
3. Knee bending exercises
   - Heel slides: 10 repetitions, 3 times daily
   - Seated knee flexion: As tolerated, 3 times daily

### Physical Therapy

- Attend all scheduled physical therapy sessions
- Follow home exercise program as prescribed
- Report any difficulties or concerns to your therapist

## **Wound Care**

### Dressing Changes

- Keep surgical dressing clean and dry
- Change dressing as directed by your surgeon
- Do not apply lotions or creams near incision
- No submersion in water (baths, pools) until cleared

### Signs of Infection to Watch For

- Increased redness or warmth around incision
- Fever over 101.5°F (38.6°C)
- Increased pain or swelling
- Foul-smelling or colored drainage
- Opening of the surgical site

## **When to Contact Your Healthcare Provider**

### Emergency Situations (Call Immediately)

- Chest pain or shortness of breath
- Severe calf pain or swelling
- Signs of infection as listed above
- Inability to move or feel the operative leg
- Fall onto the operative knee

### Non-Emergency Concerns

- Persistent nausea or inability to eat
- Uncontrolled pain despite following medication schedule
- Questions about exercises or activity levels
- Problems with prescribed medications

## **Follow-Up Care**

### Appointments

- Schedule follow-up visits as recommended
- Typical schedule:
  - 2 weeks: Wound check and suture removal
  - 6 weeks: Progress check and X-rays
  - 3 months: Functional assessment
  - 1 year: Long-term follow-up

### Long-Term Precautions

- Maintain healthy weight
- Avoid high-impact activities unless cleared
- Continue prescribed exercises for optimal recovery
- Report any new knee pain or changes in function

## Additional Notes

- Keep detailed records of progress
- Take medications only as prescribed
- Wear comfortable, supportive shoes
- Use assistive devices until cleared to discontinue
- Follow dietary recommendations to promote healing

# **Claude 3.7 Sonnet Output #3**

# **Post-Operative Care Instructions: Total Knee Arthroplasty**

## **Medication Schedule Pain Management**

- Take prescribed pain medications as directed by your surgeon
- Typical schedule includes:
  - Primary pain medication every 4-6 hours as needed
  - Anti-inflammatory medication (if prescribed) with food
  - Ice therapy for 20 minutes every 2-3 hours while awake
- Do not exceed recommended dosages
- Take medications before pain becomes severe

### **Warning Signs**

Contact your healthcare provider if you experience:

- Pain that is not controlled by prescribed medications
- Sudden increase in pain
- Pain in calf or chest
- Fever above 101.5°F (38.6°C)

### **Activity Guidelines**

### First 24-48 Hours

- Rest frequently but avoid prolonged bed rest
- Begin ankle pump exercises immediately
- Use walker or crutches as instructed by physical therapist
- No driving while taking narcotic pain medication

### Weeks 1-2

- Gradually increase walking distance using walker/crutches
- Perform prescribed exercises 2-3 times daily
- Continue using cold therapy
- Keep incision clean and dry
- No soaking in tub or swimming

### Weeks 2-6

- Progress to cane when directed by physical therapist
- Continue prescribed exercise program
- May return to light activities
- No high-impact activities
- Follow weight-bearing restrictions as specified by surgeon

### **Movement Restrictions**

- Do not pivot or twist on operated leg
- Avoid crossing legs while sitting or lying down
- Keep knee straight when resting in bed
- Use elevated toilet seat and other adaptive equipment as recommended
- No kneeling until cleared by surgeon

### **Early Mobilization Program**

### Exercises (as directed by physical therapist)

1. Ankle pumps: 10 repetitions every hour while awake
2. Quad sets: 10 repetitions, 3 times daily
3. Straight leg raises: As tolerated, 3 times daily
4. Knee extension/flexion exercises: As instructed
5. Progressive walking program: Start with 5-10 minutes, 3 times daily

### **Incision Care**

- Keep surgical dressing clean and dry
- Change dressing as directed
- Watch for signs of infection:
  - Increased redness
  - Warmth
  - Drainage
  - Odor
  - Opening of incision

### **Follow-up Care**

- First post-operative visit: _____ (to be filled by surgeon)
- Physical therapy appointments: 2-3 times per week
- Remove staples/sutures: 10-14 days post-surgery
- Regular exercise program: 6-12 weeks

### **When to Seek Immediate Medical Attention**

Call 911 or go to nearest emergency room if you experience:

- Chest pain or difficulty breathing
- Severe calf pain or swelling
- Signs of infection with fever
- Fall onto operated knee
- Inability to move or feel leg

### **Long-term Recovery Goals**

### 2-3 Months Post-Surgery

- Return to light work (if approved by surgeon)
- Independent mobility
- Knee flexion 90-110 degrees
- Full knee extension

### 6 Months Post-Surgery

- Return to most daily activities
- Improved strength and endurance
- Knee flexion 115-125 degrees
- Modified recreational activities as approved

### **Contact Information**

- Surgeon's Office: _____
- Physical Therapy: _____
- After Hours Emergency: _____

Remember: Recovery varies by individual. Follow these instructions and your healthcare team's specific guidance for optimal results.

## **DeepSeek R1 Output #1:**

# **Post-Operative Care Instructions for Total Knee Arthroplasty (Knee Replacement)**

Congratulations on completing your knee replacement surgery! Proper post-operative care is essential to ensure a smooth recovery, minimize complications, and restore function to your knee. Below are detailed instructions to guide you through the recovery process.

### **1. Pain Management**

Effective pain control is critical for your comfort and ability to participate in rehabilitation.

- Medications:
  - Take prescribed pain medications as directed by your surgeon. These may include opioids, acetaminophen, or nonsteroidal anti-inflammatory drugs (NSAIDs).
  - Do not exceed the recommended dosage, and avoid driving or operating heavy machinery while taking opioids.
  - If pain is not adequately controlled, contact your surgeon for adjustments.
- Ice Therapy:
  - Apply ice packs to the knee for 15-20 minutes every 2-3 hours during the first 48-72 hours to reduce swelling and pain.
  - Always wrap the ice pack in a towel to protect your skin.
- Elevation:
  - Keep your leg elevated above heart level when resting to reduce swelling.

### **2. Potential Side Effects**

Be aware of common side effects and know when to seek medical attention.

- Swelling and Bruising:
  - Mild to moderate swelling and bruising around the knee are normal and may persist for several weeks.
  - Use compression stockings or bandages as recommended to manage swelling.
- Fever:
  - A low-grade fever (below 101°F) is common in the first few days. However, a fever above 101°F or persistent fever could indicate an infection—contact your surgeon immediately.
- Numbness or Tingling:
  - Temporary numbness or tingling around the incision site is normal. If it persists or worsens, inform your surgeon.
- Signs of Infection:
  - Watch for redness, warmth, increased pain, or drainage from the incision. These could indicate an infection and require prompt medical attention.
- Blood Clots:
  - Symptoms of a blood clot (deep vein thrombosis) include calf pain, swelling, redness, or warmth in the leg. Shortness of breath or chest pain could indicate a pulmonary embolism—seek emergency care immediately.

### **3. Activity Restrictions**

Follow activity guidelines to protect your new knee and promote healing.

- Weight-Bearing:
  - Use a walker, crutches, or cane as instructed by your physical therapist. Gradually increase weight-bearing as tolerated.
  - Avoid putting full weight on the operated leg until cleared by your surgeon.
- Avoid Certain Movements:
  - Do not twist or pivot on the operated knee.
  - Avoid kneeling, squatting, or crossing your legs for at least 6-8 weeks.
- Driving:
  - Do not drive until your surgeon approves, typically after 4-6 weeks or when you can safely operate the vehicle.
- Work:
  - Return to work depends on your job requirements. Desk jobs may allow a return in 4-6 weeks, while physically demanding jobs may require 3-6 months.

### **4. Importance of Early Mobilization**

Early movement is crucial for recovery and preventing complications.

- Physical Therapy:
  - Begin physical therapy as soon as recommended, often within 24-48 hours after surgery.
  - Perform prescribed exercises daily to improve strength, flexibility, and range of motion.
- Walking:
  - Start walking short distances with assistance as soon as possible. Gradually increase distance and frequency as tolerated.
  - Walking helps prevent blood clots, improves circulation, and promotes healing.
- Knee Exercises:
  - Focus on exercises such as ankle pumps, heel slides, straight leg raises, and quadriceps sets to maintain mobility and strength.

### **5. Incision Care**

Proper wound care is essential to prevent infection.

- Keep the incision clean and dry. Follow your surgeon’s instructions for dressing changes.
- Do not soak the incision in water (e.g., baths, hot tubs, or swimming pools) until it is fully healed.
- Report any signs of infection, such as redness, swelling, or discharge, to your surgeon immediately.

### **6. Follow-Up Appointments**

Attend all scheduled follow-up visits with your surgeon to monitor your progress.

- Typically, follow-ups occur at 2 weeks, 6 weeks, 3 months, and 1 year post-surgery.
- X-rays may be taken to ensure proper healing and alignment of the knee implant.

### **7. Lifestyle Modifications**

Adopt healthy habits to support your recovery and long-term joint health.

- Maintain a balanced diet rich in protein, vitamins, and minerals to promote healing.
- Stay hydrated and avoid smoking, as it can impair healing.
- Gradually return to low-impact activities like swimming, cycling, or walking for exercise.

### **When to Contact Your Surgeon**

Reach out to your surgeon if you experience:

- Severe or worsening pain not relieved by medication.
- Signs of infection (fever, redness, warmth, or drainage from the incision).
- Sudden swelling, shortness of breath, or chest pain.
- Inability to bear weight or move the knee.

By following these instructions and working closely with your healthcare team, you can achieve a successful recovery and enjoy improved knee function. If you have any questions or concerns, do not hesitate to contact your surgeon or physical therapist. Wishing you a smooth and speedy recovery!

## **DeepSeek R1 Output #2:**

### **Post-Operative Care Instructions for Total Knee Arthroplasty (Knee Replacement)**

Congratulations on completing your knee replacement surgery! Proper post-operative care is essential to ensure a smooth recovery, minimize complications, and restore function to your knee. Below are detailed instructions to guide you through the recovery process.

### **1. Pain Management**

Effective pain control is critical for your comfort and ability to participate in rehabilitation.

- Medications:
  - Take prescribed pain medications (e.g., opioids, acetaminophen, or NSAIDs) as directed by your surgeon. Do not exceed the recommended dosage.
  - If you experience side effects such as nausea, constipation, or drowsiness, inform your healthcare provider.
  - Gradually transition to over-the-counter pain relievers (e.g., acetaminophen or ibuprofen) as your pain decreases.
- Non-Medication Strategies:
  - Apply ice packs to the knee for 15-20 minutes every 2-3 hours to reduce swelling and pain.
  - Elevate your leg above heart level when resting to minimize swelling.
  - Use a compression bandage or stocking if recommended by your surgeon.

### **2. Potential Side Effects**

Be aware of common side effects and know when to seek medical attention.

- Common Side Effects:
  - Swelling, bruising, and mild redness around the incision site.
  - Stiffness or discomfort in the knee, especially during activity.
  - Fatigue or mild dizziness due to anesthesia and medications.
- Warning Signs (Contact Your Surgeon Immediately):
  - Fever above 101°F (38.3°C) or chills.
  - Increased redness, warmth, or drainage from the incision site.
  - Sudden or severe swelling in the calf or thigh, which could indicate a blood clot.
  - Persistent or worsening pain not relieved by medication.
  - Shortness of breath or chest pain, which could signal a pulmonary embolism.

### **3. Activity Restrictions**

Follow activity guidelines to protect your new knee and promote healing.

- Weight-Bearing:
  - Use a walker, crutches, or cane as instructed by your physical therapist. Gradually increase weight-bearing as tolerated.
  - Avoid putting full weight on the operated leg until cleared by your surgeon.
- Movement:
  - Avoid twisting or pivoting on the operated knee.
  - Do not kneel or squat during the initial recovery period.
  - Use assistive devices (e.g., raised toilet seat, shower chair) to prevent falls.
- Driving:
  - Do not drive until your surgeon approves, typically after 4-6 weeks or when you can safely operate the vehicle.
- Work:
  - Return to work depends on your job requirements. Desk jobs may allow a return within 4-6 weeks, while physically demanding jobs may require 3-6 months.

### **4. Importance of Early Mobilization**

Early movement is crucial for restoring knee function and preventing complications.

- Physical Therapy:
  - Begin physical therapy as soon as recommended by your surgeon, often within 24-48 hours after surgery.
  - Perform prescribed exercises daily to improve strength, flexibility, and range of motion.
  - Focus on straightening (extension) and bending (flexion) the knee to regain full mobility.
- Walking:
  - Start walking short distances with assistive devices as soon as possible.
  - Gradually increase walking duration and distance as your strength improves.
- Preventing Blood Clots:
  - Perform ankle pumps and leg exercises to promote circulation.
  - Take prescribed blood thinners (if applicable) and wear compression stockings as directed.

### **5. Incision Care**

Proper wound care is essential to prevent infection.

- Keep the incision clean and dry. Avoid soaking the wound in water (e.g., baths, pools) until fully healed.
- Change dressings as instructed by your surgeon.
- Monitor for signs of infection, such as increased redness, swelling, or discharge.

### **6. Follow-Up Appointments**

Attend all scheduled follow-up visits with your surgeon and physical therapist to monitor your progress.

- Typical Follow-Up Schedule:
  - 1-2 weeks post-op: Staple or suture removal and initial wound check.
  - 6 weeks post-op: Assess knee function and progress.
  - 3-6 months post-op: Evaluate long-term recovery and activity level.

### **7. Long-Term Recovery Tips**

- Continue strengthening exercises and low-impact activities (e.g., swimming, cycling) to maintain knee health.
- Avoid high-impact activities (e.g., running, jumping) to protect the implant.
- Maintain a healthy weight to reduce stress on the knee joint.

### **Emergency Contact Information**

If you experience any concerning symptoms or have questions, contact your surgeon or healthcare provider immediately.

By following these instructions and staying committed to your rehabilitation, you can achieve the best possible outcome from your knee replacement surgery. Wishing you a smooth and successful recovery!

## **DeepSeek R1 Output #3:**

### **Post-Operative Care Instructions for Total Knee Arthroplasty (Knee Replacement)**

Congratulations on completing your knee replacement surgery! Proper post-operative care is essential to ensure a smooth recovery, minimize complications, and restore function to your knee. Below are detailed instructions to guide you through the recovery process.

### **1. Pain Management**

Effective pain control is critical for your comfort and ability to participate in rehabilitation.

- Medications:
  - Take prescribed pain medications (e.g., opioids, acetaminophen, or NSAIDs) as directed by your surgeon. Do not exceed the recommended dosage.
  - If prescribed opioids, use them only for severe pain and transition to over-the-counter pain relievers as soon as possible to avoid dependency.
  - Take stool softeners if prescribed, as opioids can cause constipation.
- Non-Pharmacological Methods:
  - Apply ice packs to the knee for 15-20 minutes every 2-3 hours to reduce swelling and pain.
  - Elevate your leg above heart level when resting to minimize swelling.
- Monitor for Side Effects:
  - Notify your surgeon if you experience severe pain, redness, warmth, or swelling that worsens, as these could indicate infection or blood clots.

### **2. Potential Side Effects**

Be aware of common side effects and complications, and know when to seek medical attention.

- Common Side Effects:
  - Swelling and bruising around the knee (normal for the first few weeks).
  - Mild to moderate pain, especially with movement.
  - Stiffness or difficulty bending the knee initially.
- Warning Signs (Contact Your Surgeon Immediately):
  - Fever (>101°F) or chills, which may indicate infection.
  - Severe pain not relieved by medication.
  - Redness, warmth, or drainage from the incision site.
  - Sudden swelling or calf pain, which could signal a blood clot (deep vein thrombosis).

### **3. Activity Restrictions**

To protect your new knee and promote healing, follow these activity guidelines:

- Weight-Bearing:
  - Follow your surgeon’s instructions regarding weight-bearing. Most patients can bear weight as tolerated with the assistance of a walker or crutches initially.
  - Gradually transition to a cane as your strength and balance improve.
- Avoid Certain Movements:
  - Do not twist or pivot on your operated leg.
  - Avoid high-impact activities (e.g., running, jumping) until cleared by your surgeon.
  - Do not kneel on the operated knee for at least 6-8 weeks.
- Driving:
  - Do not drive until your surgeon approves, typically after 4-6 weeks or when you can safely operate the vehicle.
- Work:
  - Return to work depends on your job type. Desk jobs may allow a return in 4-6 weeks, while physically demanding jobs may require 3-6 months.

### **4. Importance of Early Mobilization**

Early movement is crucial for recovery and preventing complications.

- Physical Therapy:
  - Begin physical therapy as soon as recommended, often within 24-48 hours after surgery.
  - Perform prescribed exercises daily to improve strength, flexibility, and range of motion.
  - Focus on straightening (extension) and bending (flexion) the knee.
- Walking:
  - Start walking short distances with assistive devices (walker, crutches) as soon as possible.
  - Gradually increase walking distance as your strength improves.
- Preventing Stiffness:
  - Avoid prolonged periods of inactivity. Move your ankle and foot frequently to promote circulation.
  - Use a continuous passive motion (CPM) machine if prescribed.

### **5. Wound Care**

Proper care of your incision site is essential to prevent infection.

- Keep the Incision Clean and Dry:
  - Follow your surgeon’s instructions for dressing changes and showering.
  - Do not soak the incision in water (e.g., baths, swimming) until fully healed.
- Monitor for Infection:
  - Watch for signs of infection, such as redness, swelling, warmth, or drainage from the incision.

### **6. Follow-Up Appointments**

Attend all scheduled follow-up visits with your surgeon to monitor your progress.

- Typical Schedule:
  - First follow-up: 1-2 weeks post-op for wound check and stitch/staples removal.
  - Subsequent visits: 6 weeks, 3 months, and 1 year post-op.

### **7. Long-Term Recovery**

Full recovery can take 6-12 months, but most patients experience significant improvement within 3-6 months.

- Continue Exercises:
  - Maintain a regular exercise routine to strengthen the knee and surrounding muscles.
  - Low-impact activities like swimming, cycling, and walking are encouraged.
- Lifestyle Modifications:
  - Maintain a healthy weight to reduce stress on the new knee.
  - Use assistive devices (e.g., cane, handrails) as needed for safety.

### **8. Emotional Support**

Recovery can be challenging, both physically and emotionally. Seek support from family, friends, or support groups to stay motivated.

If you have any questions or concerns during your recovery, do not hesitate to contact your surgeon or healthcare team. Your commitment to following these instructions will greatly contribute to a successful outcome. Wishing you a smooth and speedy recovery!

## **OpenAI GPT-4o Output #1:**

### **Post-Operative Care Instructions for Total Knee Arthroplasty (Knee Replacement)**

**1. Pain Management:**

- Medications: You will be prescribed pain medications to manage your post-surgical pain. These may include:
  - Opioids (e.g., oxycodone, hydrocodone): Take as prescribed. Use them for short-term relief only.
  - Nonsteroidal Anti-Inflammatory Drugs (NSAIDs, e.g., ibuprofen): These help reduce inflammation and pain.
  - Acetaminophen: Can be used alongside NSAIDs if directed by your doctor.
  - Local anesthetics or nerve blocks may also be provided for temporary pain relief.
- Ice and Elevation: Apply ice to your knee for 20-minute intervals, especially in the first few days, to reduce swelling and pain. Keep your leg elevated (above the level of your heart) to further reduce swelling.
- Stay Ahead of Pain: Take your medications as scheduled, not just when you feel pain. Gradually reduce the use of opioids as your pain improves.

**2. Potential Side Effects:**

- Pain Medications:
  - Opioid side effects may include drowsiness, constipation, nausea, and dizziness. To prevent constipation, drink plenty of fluids, eat fiber-rich foods, and consider using a stool softener.
  - NSAIDs can cause stomach upset or increase the risk of bleeding. Take them with food and avoid if you have a history of ulcers or gastrointestinal issues.
- Swelling and Bruising: Swelling around the knee and lower leg is common. It should gradually improve over the next few weeks. However, if swelling increases significantly or if you notice redness and warmth, contact your doctor.
- Blood Clots: There's a risk of developing blood clots after surgery. You may be prescribed blood thinners (e.g., aspirin or anticoagulants). Be alert to symptoms of deep vein thrombosis (DVT), such as severe leg swelling or calf pain, and contact your healthcare provider immediately if you notice them.
- Infection: Signs of infection include increased redness, warmth, unusual pain, or drainage from the incision site. A low-grade fever is common, but contact your doctor if your temperature exceeds 101°F.

**3. Activity Restrictions:**

- Weight-Bearing and Walking: Follow your surgeon's instructions regarding weight-bearing. You may need crutches or a walker initially to support you. Gradual walking is encouraged to aid recovery, but avoid overexerting yourself in the first few days.
- Avoid Strenuous Activities: Refrain from high-impact activities such as running, jumping, or heavy lifting for at least 6-12 weeks or until cleared by your surgeon.
- Physical Therapy: You will begin physical therapy shortly after surgery. Consistently follow the prescribed exercises, as these are critical for regaining strength, flexibility, and knee function. Attending your physical therapy sessions is vital for a successful recovery.
- Driving: Do not drive until your doctor clears you, which may be several weeks depending on your progress. Avoid driving while taking opioids or other medications that can impair your alertness.

**4. Importance of Early Mobilization:**

- Prevent Blood Clots: Early movement, such as walking or doing gentle foot and ankle exercises, helps prevent blood clots in your legs.
- Improving Range of Motion: Early mobilization promotes flexibility in the knee and prevents stiffness. Start gentle bending and straightening exercises as directed by your physical therapist.
- Quicker Recovery: Gradual and consistent movement aids the healing process and improves overall outcomes. Follow your physical therapy plan closely, as it is designed to help you regain normal function.
- Posture and Gait: Pay attention to posture and avoid limping to protect your healing knee. Your therapist will help guide you on proper walking techniques.

**5. Follow-Up Care:**

- Attend all scheduled follow-up appointments with your surgeon. X-rays may be taken to ensure proper healing of the implant.
- Report any concerning symptoms, such as persistent or worsening pain, signs of infection, or difficulties with mobility.

These care instructions are aimed at helping you recover safely and effectively after knee replacement surgery. Consistently following these guidelines will improve your recovery and long-term outcomes.

## **OpenAI GPT-4o Output #2:**

### **Post-Operative Care Instructions for Total Knee Arthroplasty (Knee Replacement)**

**1. Pain Management:**

- Medications:
  - Prescribed pain relievers: Take as instructed. This may include a combination of opioids (e.g., oxycodone or hydrocodone) for short-term use, and non-steroidal anti-inflammatory drugs (NSAIDs), such as ibuprofen or naproxen.
  - Tylenol (acetaminophen): Can be used for additional pain relief.
  - Avoid overuse of opioids: These are intended for short-term pain control due to their risk of dependence. Use the lowest dose possible, and taper off as soon as pain improves.
  - Ice therapy: Apply an ice pack to the knee for 15-20 minutes every 1-2 hours during the first 72 hours to reduce pain and swelling.
  - Elevation: Keep your leg elevated (above heart level) while resting to help reduce swelling and discomfort.
  - Nerve block or local anesthesia: If a nerve block was administered during surgery, you might feel numbness for a few hours to days. This can help with pain but avoid putting weight on the leg until sensation returns.

**2. Potential Side Effects:**

- Pain and Swelling: Common after surgery and should gradually improve. If the swelling worsens or does not improve, contact your doctor.
- Bruising: It is normal to have bruising around the knee, thigh, or lower leg.
- Nausea or Drowsiness: You may feel drowsy or nauseous from anesthesia or pain medications. Drink fluids and eat small, light meals to help with this.
- Constipation: Pain medications can cause constipation. Increase your fiber intake and stay hydrated. You may also use a stool softener as recommended.
- Infection: Signs of infection include increased redness, warmth, drainage, or fever above 100.4°F. Contact your healthcare provider if these occur.
- Blood Clots (Deep Vein Thrombosis): Symptoms include swelling, pain, or redness in your calf or thigh. You may be prescribed blood thinners to prevent clots. Wear compression stockings as recommended.

**3. Activity Restrictions and Early Mobilization:**

- Weight-Bearing: Follow your surgeon’s instructions on how much weight you can put on your leg. You may start with a walker or crutches, gradually increasing weight-bearing as tolerated.
- Movement and Exercise:
  - Physical Therapy: Begin physical therapy as soon as recommended (usually the day of or day after surgery). Therapy will focus on range-of-motion exercises, strengthening, and improving mobility.
  - Home exercises: Perform your home exercise program regularly to strengthen the knee, prevent stiffness, and promote circulation.
- Avoid High-Impact Activities: Avoid running, jumping, and heavy lifting until cleared by your surgeon.
- Stairs: Be cautious when climbing stairs. Use handrails and support as needed. Physical therapy will help you practice safe stair climbing.
- Driving: Do not drive until cleared by your doctor, usually after 4-6 weeks when you can comfortably control the vehicle.

**4. Importance of Early Mobilization:**

- Prevents Complications: Early mobilization helps prevent blood clots, improve circulation, and reduce swelling. It also helps with muscle strength and flexibility.
- Improves Recovery: Starting physical activity early can lead to a faster and more successful recovery. It’s important to follow your therapy program diligently.
- Knee Flexion and Extension: Regaining full range of motion in your knee is critical for daily activities. Focus on bending and straightening your knee as instructed by your therapist.
- Walking Aids: Use a walker, crutches, or a cane as advised. These aids will help you move safely while maintaining proper balance.

**5. Follow-Up Care:**

- Post-Surgical Appointments: Attend all follow-up visits with your surgeon. These appointments will monitor your healing progress and ensure that there are no complications.
- Incision Care: Keep the incision clean and dry. Follow your doctor’s instructions on when it is safe to shower. Watch for signs of infection.
- Long-Term Care: It may take several months to fully recover. Continue strengthening and stretching exercises to improve long-term function. Avoid activities that put excessive strain on your knee.

Following these post-operative care instructions will help ensure a smooth recovery after total knee replacement surgery.

## **OpenAI GPT-4o Output #3:**

### **Post-Operative Care Instructions for Total Knee Arthroplasty (Knee Replacement)**

**1. Pain Management**

- Medications: You will be prescribed pain medications to manage discomfort after surgery. These may include:
  - Opioids (e.g., oxycodone, hydrocodone) for short-term use. Take as prescribed and only when needed to avoid dependence.
  - NSAIDs (e.g., ibuprofen) to reduce inflammation and pain. Avoid overuse to protect your stomach and kidneys.
  - Acetaminophen (Tylenol) can also be taken to reduce pain.
  - Ice Therapy: Apply ice packs to the knee for 20 minutes every hour while awake to reduce swelling and pain. Make sure to place a cloth between the ice pack and skin.
  - Nerve Block: If a nerve block was used during surgery, it may provide pain relief for several hours or days. Once it wears off, follow the prescribed pain medication schedule.

**2. Potential Side Effects**

- Pain and Swelling: It is normal to experience pain, swelling, and stiffness in the knee for a few weeks post-surgery.
- Nausea and Constipation: Pain medications, especially opioids, may cause nausea or constipation. To manage these:
  - Take medications with food.
  - Stay hydrated and eat a high-fiber diet.
  - Use a stool softener or laxative if recommended by your doctor.
- Blood Clots (Deep Vein Thrombosis - DVT): You will likely be prescribed blood thinners to reduce the risk of blood clots. Signs of DVT include swelling, redness, and pain in the calf. If you experience these symptoms, contact your doctor immediately.
- Infection: Watch for signs of infection, such as increased redness, warmth, or drainage from the incision, or fever above 100.4°F. Keep the incision clean and dry.

**3. Activity Restrictions**

- Weight-Bearing: Depending on your surgeon’s instructions, you may be allowed to put some weight on the operated leg with assistance (walker or crutches). Always follow your surgeon’s guidance.
- Driving: You should not drive until cleared by your doctor, typically 4-6 weeks after surgery and once you are no longer taking opioid pain medications.
- Physical Activity: Avoid high-impact activities (e.g., running, jumping) or heavy lifting (over 10 pounds) until cleared by your doctor. However, walking short distances is encouraged to prevent blood clots and improve circulation.
- Bathing: You may shower once your surgeon approves, but avoid soaking the incision in baths, hot tubs, or pools until it is fully healed, usually after 3-4 weeks.

**4. Importance of Early Mobilization**

- Physical Therapy: Early mobilization is key to a successful recovery. You will start physical therapy shortly after surgery to:
  - Improve range of motion.
  - Strengthen muscles around the knee.
  - Prevent stiffness and scar tissue formation.
  - Follow the exercises prescribed by your physical therapist diligently.
- Walking: Start walking short distances with the assistance of a walker or crutches as soon as possible. Gradually increase the distance each day.
- Leg Elevation: Elevate your leg while resting to reduce swelling. Keep the leg above the level of your heart when possible.

**5. Follow-Up Care**

- Appointments: Attend all follow-up appointments with your surgeon. X-rays may be taken to ensure proper healing of the knee joint.
- Knee Brace/Support: If your surgeon has provided a knee brace or support, wear it as directed, especially during walking or physical therapy.
- Long-Term Recovery: Full recovery can take 3-6 months. During this time, continue with physical therapy, gradually increasing activity, and monitoring for any complications.

If you experience unusual pain, swelling, or any concerning symptoms, contact your healthcare provider immediately. Your recovery progress will depend on following these instructions carefully and staying active within the prescribed limits.
